# Renal Reabsorption of Folates: Pharmacological and Toxicological Snapshots

**DOI:** 10.3390/nu11102353

**Published:** 2019-10-02

**Authors:** Sophia L Samodelov, Zhibo Gai, Gerd A Kullak-Ublick, Michele Visentin

**Affiliations:** 1 Department of Clinical Pharmacology and Toxicology, University Hospital Zurich, University of Zurich, 8006 Zurich, Switzerland; Sophia.Samodelov@usz.ch (S.L.S.); gerd.kullak@usz.ch (G.A.K.-U.); michele.visentin@usz.ch (M.V.); 2 Mechanistic Safety, CMO & Patient Safety, Global Drug Development, Novartis Pharma, 4056 Basel, Switzerland

**Keywords:** acute kidney injury, folate, folate receptor, folic acid, nephrotoxicity, renal reabsorption

## Abstract

Folates are water-soluble B9 vitamins that serve as one-carbon donors in the de novo synthesis of thymidylate and purines, and in the conversion of homocysteine to methionine. Due to their key roles in nucleic acid synthesis and in DNA methylation, inhibiting the folate pathway is still one of the most efficient approaches for the treatment of several tumors. Methotrexate and pemetrexed are the most prescribed antifolates and are mainly used in the treatment of acute myeloid leukemia, osteosarcoma, and lung cancers. Normal levels of folates in the blood are maintained not only by proper dietary intake and intestinal absorption, but also by an efficient renal reabsorption that seems to be primarily mediated by the glycosylphosphatidylinositol- (GPI) anchored protein folate receptor α (FRα), which is highly expressed at the brush-border membrane of proximal tubule cells. Folate deficiency due to malnutrition, impaired intestinal absorption or increased urinary elimination is associated with severe hematological and neurological deficits. This review describes the role of the kidneys in folate homeostasis, the molecular basis of folate handling by the kidneys, and the use of high dose folic acid as a model of acute kidney injury. Finally, we provide an overview on the development of folate-based compounds and their possible therapeutic potential and toxicological ramifications.

## 1. Introduction

Folates are water-soluble B9 vitamins that consist of a pteridine moiety, a para-aminobenzoate group and a tail of glutamate residues, which is important for the intracellular retention of folates. The redox state of pteridine defines oxidized (folic acid) and reduced folates (tetrahydrofolate, THF), with the latter being the naturally occurring folates. In the cell, folates serve as one-carbon donors and provide a methylene group for the de novo synthesis of thymidylate from deoxyuridylate and two formate groups for the de novo synthesis of the purines. Thus, they are essential for DNA and RNA synthesis and, in turn, for cell proliferation. Additionally, 5-methylTHF donates its methyl group for the conversion of homocysteine to methionine. The key role of folates in homocysteine catabolism makes homocysteinemia a reliable indicator of systemic folate status, as well as making folate (and methylcobalamin) supplementation the standard approach for lowering homocysteine blood levels [1,2]. Folate deficiency is associated with profound disturbances that affect, in particular, highly proliferating tissues, such as the bone marrow and the gastrointestinal mucosa, as well as the developing central nervous system of infants [3]. Due to its key role in the synthesis of DNA and RNA building blocks and in DNA methylation, blocking the folate pathway remains one of the most efficient approaches for the treatment of several tumors. Methotrexate and pemetrexed are the most prescribed antifolates and are often used in the treatment of acute myeloid leukemia, osteosarcoma, non-small cell lung cancer, and mesothelioma [4].

The major dietary folate in nature, 5-methyltetrahydrofolate (5-methylTHF) in polyglutamylated forms, is found in a wide variety of foods, particularly in green leafy vegetables, especially when consumed fresh and raw. In fact, a considerable amount of folates can be lost during harvesting, storage and cooking [5,6]. Folic acid, the oxidized form, does not occur naturally and, as it is not the physiological form, it is practically absent in the plasma, with the exception of the populations of those countries that have embraced folic acid food fortification strategies, such as those in North America. In these populations, folic acid accounts for about 5% of the total circulating folate pool [7]. In these individuals, the overall plasma level of folates is about 40 nM, roughly double the level measured in the populations not subjected to the food fortification or other types of folate supplementation [7,8]. Folates are absorbed primarily in the small intestine by the electrogenic symporter proton-coupled folate transporter (PCFT, *SLC46A1*) [9]. Loss of function mutations of the PCFT cause hereditary folate malabsorption (HFM), an autosomal recessive disorder associated with severe hematological and neurological deficits [3,10,11]. The role of PCFT in HFM was confirmed by phenotyping the Pcft^−/−^ mouse. These animals are apparently normal at birth, likely due to an adequate maternal folate storage. However, by four weeks of age, this supply is depleted and the pups show systemic folate deficiency with macrocytic anemia and pancytopenia, very much like the children with HFM [12]. Non-genetic factors that can alter PCFT expression and function are alcohol abuse and vitamin D3 supplementation, which represses and induces PCFT expression, respectively [13,14,15]. Upon intestinal absorption, folates circulate in the serum freely, bound to a small pool of soluble high-affinity folate binding protein (FBP), or loosely associated to albumin or other soluble proteins [16,17]. The cellular transport of folates from the blood into the cells is mainly mediated by the anion exchanger reduced folate carrier (RFC, *SLC19A1*) [18]. Loss of function mutations in the *SLC19A1* gene are embryonic lethal [19].

Normal levels of folates in the blood are maintained not only by proper dietary intake and intestinal absorption, but also by an efficient renal reabsorption mainly mediated by the glycosylphosphatidylinositol- (GPI) anchored protein folate receptor α (FRα) [20]. FRα is highly expressed in several epithelial malignancies, hence considered an ideal target for selective delivery of drug cargos [21]. Nonetheless, as FRα-mediated transport is particularly relevant in proximal tubules, kidneys inevitably represent the target site of toxicity. As part of the Special Issue “Dietary Folate and Human Health”, this review describes the role of the kidneys in folate homeostasis, the molecular basis of folate handling by the kidneys, and the therapeutic and toxic features of folate accumulation in the renal parenchyma. Finally, we provide an overview of folate-conjugated drugs viewed from the kidney standpoint.

## 2. Mechanisms of Folate Tubular Reabsorption 

**Figure 1:**
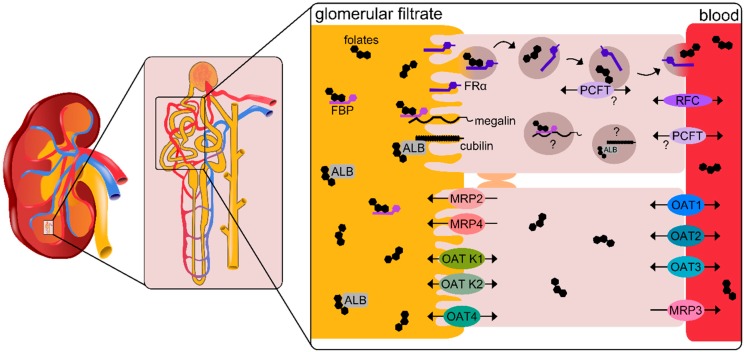
Renal reabsorption of folates from glomerular filtrate in the proximal tubules under physiological conditions is postulated to mainly rely on the high-affinity folate receptor FRα through a process of transcytosis to the basolateral membrane into the blood. Possible roles of megalin, which binds soluble folate binding proteins (FBP), and cubilin, which binds albumin (ALB), for uptake of folates under conditions of folate deficiency over additional endocytic mechanisms has also been proposed. The folate-specific transporters reduced folate carrier (RFC) and proton-coupled folate transporter (PCFT) are highly expressed in the proximal tubule, with known expression of RFC at the basolateral membrane and unknown location and function of PCFT in the context of renal folate transport. Additional low-affinity, high-capacity folate transporters expressed at the apical membrane include the multidrug resistance-associated proteins (MRPs) efflux pumps 2 and 4 and the organic anion transporters (OAT) K1, K2, and 4. At the basolateral membrane, OAT1, 2, and 3 as well as the efflux pump MRP3 may likewise contribute to renal folate transport and general folate homeostasis.

As folates are water-soluble vitamins of small size (5-methylTHF in its monoglutamylated form has an exact mass of 459.19 g/moL), they are freely filtered by the glomerulus [22]. Under normal folate levels, little renal excretion and high reabsorption over the brush border membrane of the proximal tubule cells can be observed [22,23,24]. Much research has focused on the mechanisms of renal reabsorption of folates and has uncovered receptor-mediated endocytosis followed by transcytosis, as well as the postulation of facilitated transport over several transmembrane spanning transporters, identified over their ability to recognize antifolates as substrates. The concerted action of these several membrane proteins is decisive to ensure the recycling and conservation of folates (Figure 1).

A well-described mechanism of receptor-mediated endocytosis involves the membrane bound FBP, GPI-anchored high-affinity folate receptor FOLR1 (FRα) [25]. FRα is highly expressed on the luminal/apical side of proximal tubule epithelial cells and has a high affinity for the biologically relevant 5-methylTHF (K_D_ = 5–10 nM) as well as for several other reduced folates that are usually present only in trace amounts in the blood stream [26]. Fully oxidized folic acid, currently particularly relevant in the context of the development of folic acid-conjugated drugs (see below), is bound with an even higher affinity (K_D_ < 1 nM) by FRα [26]. Binding of free folates to this receptor leads to cell membrane invagination, endosomal acidification, ligand release, and recycling of the receptor to the outer leaflet [27]. FRα cycles between an acid labile, surface accessible, ligand binding conformation, and an acid resistant, internalized, ligand releasing conformation, remaining membrane-bound throughout [26]. It was initially postulated that FRα, as assumed for other GPI-anchored proteins, becomes concentrated in membrane caveolae upon crosslinking caused by ligand binding, leading to potocytosis (receptor-mediated endocytosis, with release from caveolae directly into the cytosol upon invagination) [28,29]. However, studies showing diffuse distribution of GPI-anchored proteins [30] and lack of localization of FRα in caveolae, but rather in clathrin-coated endocytic pits [31], have led to the reevaluation of the exact mechanism of endocytosis of folates over FRα, with the postulation of receptors being constantly clustered by colocalization in lipid rafts [32]. Intravital imaging of intact rat kidneys following the endocytosis of a pH insensitive folic acid-fluorescent dye conjugate showed folate endocytosis at the brush border membrane, localization to lysosomal pools, as well as complete transcytosis to the basal membrane [33]. Interestingly, no release to the cytosol of the folic acid conjugates used in this study was observed. This suggests that, during renal reabsorption, most folates are trapped in endosomal vesicles and take the transcytotic pathway, with minimal release to the cytosol of proximal tubule cells. 

A possible interaction of FRα with other general broad-ligand endocytosis-mediating receptors megalin (LRP2), and possibly in concert with cubilin, has also been proposed [16,34]. Megalin and cubilin are two structurally different surface proteins: megalin is a member of the low-density lipoprotein (LDL)-receptor family and has a large extracellular domain with several ligand-binding regions, a transmembrane domain, and a short cytoplasmic tail; cubilin has several binding domains as well, but no obvious transmembrane domain [35]. Both megalin and cubilin, which notably bind folate-associated soluble serum FBP and albumin, respectively, are highly expressed in the proximal tubule as proteins responsible for the uptake of a broad range of albumin and protein-bound substrates. The relative physiological impact of these endocytosis-mediating receptors for renal folate uptake into proximal tubule cells at the luminal/apical side remains unclear, although it is generally established that FRα plays a large, if not the largest, role in renal reabsorption of folates under normal circumstances. A targeted gene knockout of folr1 in the kidneys of mice was shown to lead to lower plasma folates and increased renal clearance in folr1^−/−^ mice both on normal and reduced folate diets, indicating increased loss of folates via excretion [20]. Crude kidney membrane extracts from these mice were unable to bind ^3^H-folic acid and bound little unlabeled folic acid when incubated in excess concentrations. Under normal dietary conditions, folate clearance was found to be approximately 100% that of creatinine clearance in knockout mice, indicating no reabsorption of folates under loss of kidney *folr1* gene. Interestingly, a low folate diet led to only a 20% folate clearance compared to creatinine clearance in *folr1^−/−^* mice [20]. This leads to the speculation that other processes may play a larger role in renal folate reabsorption under folate deficiency, such as the uptake of FBP-bound folate reabsorbed over megalin in the proximal tubule. The small pool of circulating serum FBP secreted by hemopoietic cells has been shown to be constantly saturated with folate under normal conditions [36]. This would have implications for increased megalin-mediated renal reabsorption when serum folate levels are low, where a larger relative amount of serum folate is bound to high-affinity soluble FBP and thus subject to megalin-mediated endocytosis instead of over FRα. 

Aside from endocytic mechanisms of folate reabsorption, endocytosis-independent reabsorption of 5-methylTHF in proximal tubule cells is supported by transport studies performed in primary human proximal tubule cells cultured on membrane inserts to exhibit differentiated apical and basolateral domains [37]. In these studies, most of the apical membrane uptake of 5-methylTHF could be inhibited by addition of the microtubule depolymerizer colchicine. Moreover, in the presence of folic acid (for which FRα has a much higher affinity towards than for 5-methylTHF), 5-methylTHF binding to the apical membrane was completely abolished, while transport of 5-methylTHF across the apical membrane was diminished only by half. The remaining transport activity could be blocked by the addition of the anion exchange inhibitor, probenecid, indicating additional anion-dependent uptake of folates at the apical membrane [37]. Several membrane-spanning transporters are highly expressed in the proximal tubule and may contribute, to varying extents, to folate vectorial transport. Specific transporters of folate include, on the basolateral membrane, RFC and PCFT [38,39,40]. It has been postulated that RFC is responsible for the transport of folate bidirectionally across the basal membrane of proximal tubule cells [41,42]. The exact location of expression and the role of the PCFT in renal reabsorption of folates is unclear. However, because PCFT transports folates with an optimum at acidic pH, a direct contribution to the uptake of folates in the kidney is unlikely. Nonetheless, it may be relevant in the tubular secretion of pemetrexed, which is transported by PCFT also at neutral pH [43,44]. For other cell types, such as retinal cells, because FRα and PCFT colocalize in endosomal compartments during folate uptake, it has been hypothesized that, upon releasing of folates from the receptor, PCFT facilitates their exit from the endosome into the cytosol by exploiting the existing proton gradient across the endosomal membrane [45]. However, the activity of FRα and PCFT are likely uncoupled in tubular reabsorption of folates because of the transcytotic nature of FRα-mediated transport in the kidney. Interestingly, mRNA and protein levels of RFC and PFCT have been shown to increase in the kidneys of rats under folate-deficient diets, whereas FRα mRNA seemed unchanged in comparison to control diet rats [46]. This may again be an indication for FRα being largely responsible for general folate homeostasis under normal serum folate concentrations with other, possibly more rapid transport mechanisms, playing an increased role in folate conservation under folate deficiency.

Besides folate-specific transporters, the contribution to renal folate reabsorption of several other organic anion transporters (OATs) as well as ATP-binding cassette (ABC) multidrug resistance-associated protein (MRPs) efflux pumps, expressed in the proximal and distal tubules, can also not be ruled out, due to their affinity for and transport of folates and/or antifolates [43,47]. In vitro studies with MRP-expressing cells identified several members of the family being able to transport antifolates [48,49,50,51,52,53]. In a renal setting, MRP2 and 4 (ABCC2 and 4) [50,54] are expressed at the apical brush border membrane of rat and mouse proximal tubule cells [55,56], functioning as efflux transporters of methotrexate [48,50]. MRP3 (ABCC3), also a methotrexate exporter, is expressed in the basolateral membranes of human distal tubules [57]. OAT-K1 and OAT-K2 of the solute carrier 21 (SLC21) transporter family are expressed at the apical brush border membrane and transport both methotrexate and 5-methylTHF [58]. SLC22 family members OAT1, OAT2, and OAT3 are expressed at the basolateral side of rat renal tubules [54,59,60], with OAT4 found in the apical one [61]. However, human OAT1 has been shown not to transport methotrexate [49,62] Whether these low-affinity, albeit high-capacity, transporters play a role in endogenous renal 5-methylTHF conservation is unclear. The high affinity binding to endogenous folates by FBPs, both for membrane-bound FRα and serum FBP, suggests that folate homeostasis by the reabsorption of the endogenous 5-methylTHF under non-pathological conditions is likely mediated primarily by receptor-mediated endocytosis/transcytosis. 

## 3. Folic Acid Treatment in Patients with Chronic Kidney Disease (CKD)

Folic acid supplementation is advocated by physicians for patients with chronic kidney disease (CKD), especially those under hemodialysis, to prevent nutritional deficiency and to facilitate hematopoiesis, though its beneficial effect on disease progression is unclear [63,64]. CKD is a global health burden with a worldwide prevalence of 11% [65]. Homocysteine concentration is elevated in the plasma of patients with CKD disease undergoing hemodialysis and positively correlates with their cardiovascular risk, though whether hyperhomocysteinemia is a risk factor of cardiovascular disease or just a bystander is still controversial [66,67,68]. Some small-sized studies in hemodialysis populations have suggested a potential benefit from folic acid and methylcobalamin (vitamin B12) administration to lower homocysteine levels and cardiovascular risk [67,69]. Nonetheless the bulk of data in both the general population [70,71] and patients on hemodialysis [72,73] suggest that lowering homocysteine levels with folic acid and B vitamins does not reduce the risk of cardiovascular events. The homocysteinemia in kidney and end-stage renal disease (HOST) trial randomized 2056 adults with an estimated glomerular filtration rate (eGFR) of less than 30 mL/min/1.73 m^2^ (*n* = 1305) or end stage kidney disease (*n* = 751) to a regimen of folic acid and other B group vitamins or placebo [73]. Despite an overall 25.5% reduction in plasma homocysteine level in the folic acid group, there was no difference in the rate of cardiovascular events and mortality between the two groups. The folic acid for vascular outcome reduction in transplantation (FAVORIT) trial randomized 4110 kidney transplant recipients with elevated homocysteine levels and an eGFR of 30 mL/min/1.73 m^2^ or greater to either a multivitamin tablet, containing folic acid, vitamin B6, and vitamin B12, or to a low-dose vitamin B6/B12 regimen [74]. Despite the expected reduction in homocysteine levels in the treated group, there was no difference in a composite cardiovascular endpoint. However, the power of individual studies to detect a benefit may have been reduced by the widespread pre-study folic acid fortification in dialysis recipients, because a meta-analysis of seven trials involving around 3800 patients suggested a benefit to folic acid supplementation and lowering of homocysteine levels [75]. 

## 4. Folic Acid-Induced Acute Kidney Injury Model

Though folate supplementation has been shown to have multiple benefits for the organism, high, suprapharmacological doses of folic acid do harm the kidneys. Folic acid-induced renal hypertrophy was first observed in rats in the 1960s, though it has never been reported in humans [76,77,78]. Ever since, the mechanisms underlying folic acid-induced kidney injury have been investigated, partially characterized, and, to this day, considered a valuable animal model to study acute kidney injury onset, progression, and possible protective strategies [78,79,80,81,82]. 

Luminal crystal formation in the mouse kidney has been reported upon injection of high dose folic acid (250 mg/kg) [83]. Additionally, folic acid may directly harm the tubular epithelium [84,85]. Besides the rapid increases of serum creatinine and blood urea nitrogen (BUN) levels, a single dose injection of folic acid reduces tubular functions and increases kidney fluid content probably due to an overall impairment in solute handling, secondary to the inhibition of the Na^+^/K^+^ ATPase activity [85,86]. High-resolution proteomic analysis of the kidneys of mice treated with high dose folic acid showed that the involvement of the glutamatergic signaling system, renin-angiotensin aldosterone system, reactive oxygen species (ROS) production, pro-apoptotic p53 pathway, and the noncanonical NF-kB pathways, which triggers kidney injury through the promotion of apoptosis and inflammation [87,88].

Folic acid-induced nephrotoxicity presents with features of ferroptosis and necroptosis [89]. Ferroptosis is an iron-dependent programmed cell death characterized by lipid peroxidation, primarily polyunsaturated fatty acids (PUFAs), initiated through non-enzymatic (Fenton reactions) and enzymatic mechanisms (lipoxygenases) [90]. Ferroptosis appears to be pivotal in folic acid-induced kidney injury as animals treated with folic acid and ferrostatin-1, a ferroptosis inhibitor, showed limited histologic injury, oxidative stress, and macrophage infiltration and displayed preserved renal function as compared with those solely treated with folic acid [91]. Necroptosis is a programmed necrosis characterized by reduced glutathione metabolism as well as upregulation of necroptosis mediators, such as receptor-interacting protein kinase 3 (RIPK3) or mixed lineage domain-like protein (MLKL) [90,91,92]. In contrast, genetic deficiency of necroptosis mediators, did not preserve renal function but reduced inflammation at early time points (48 h) [91], though necroptosis may be the primary cell death mechanism at later time points (72–96 h) [93]. It seems that ferroptosis is the primary cause of folic acid-induced acute kidney injury with necroptosis-mediated inflammation that further worsens the damage. 

Besides acute kidney injury, high dose folic acid also promotes kidney fibrosis [94]. The most important contributors to the development of tubulointerstitial fibrosis are renin activity [95], the transforming growth factor beta (TGFb) signaling pathway [96], and mitochondrial biogenesis [97], which may contribute to tissue hypoxia, fibrogenesis, endoplasmic reticulum stress, and ROS production [94,98]. Downregulation of vascular epidermal growth factor (VEGF) may be functionally implicated in the progressive attrition of peritubular capillaries and tissue hypoxia, as shown in mouse folic acid nephropathy [99].

## 5. Nephrotoxicity of Folate-Conjugated Chemotherapy and Radiotherapy

**Figure 2:**
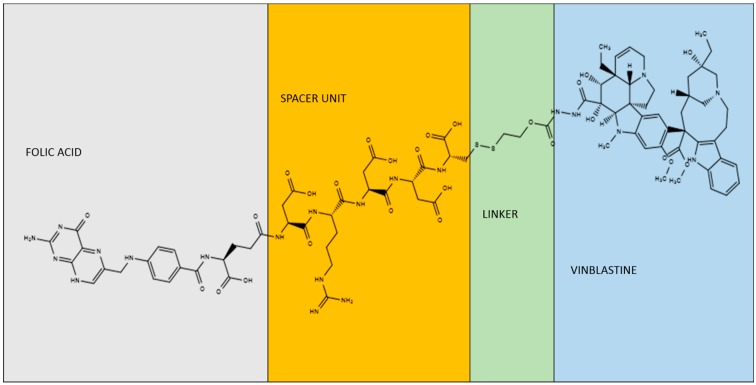
Vintafolide (EC145). The chemical structure was generated using the open access software Chemspider (http://www.chemspider.com/). Vintafolide structure was downloaded from the DrugBank (https://www.drugbank.ca/drugs/DB05168).

Besides the proximal tubules of the kidneys, only few normal tissues have shown some degree of FRα protein, specifically the choroid plexus, lung, and placenta [100,101,102,103]. Because many cancers overexpress FRα, the concept of using folic acid to deliver attached antiproliferative and/or cytotoxic drugs selectively to FRα-expressing tumor cells has received considerable attention. The most advanced compound in drug development is Vintafolide (EC145), which consists of a folic acid molecule attached via a self-immolative disulfide-based linker system to the lipophilic desacetylvinblastine hydrazide (Figure 2), a Vinca alkaloid that, in its free form, failed phase I clinical trials due to severe toxicity [104,105]. Vintafolide in combination with pegylated liposomal doxorubicin was compared to pegylated liposomal doxorubicin alone in a randomized phase II trial in women with recurrent platinum-resistant ovarian cancer. Median progression-free survival (PFS) of the patients treated with Vintafolide + pegylated liposomal doxorubicin was significantly longer (5.0 vs. 2.7 months) than that of patients treated with the pegylated liposomal doxorubicin-alone. The benefit was even greater for the patients with tumors with the highest percentage of FRα-positive cells. Consistently, cancers that poorly expressed FRα were resistant to Vintafolide treatment [106].

Because neither RFC nor PCFT, the transporters responsible for folate uptake in most epithelia, recognize the folic acid conjugates as substrates, most normal tissues are insensitive to these compounds. Surprisingly, despite the fact that proximal tubule cells abundantly express FRα, also kidney toxicity has never been observed in preclinical and clinical drug safety assessment of Vintafolide [104,105,107,108,109]. It is predicated that the lack of nephrotoxicity is due to the different mechanism of FRα-mediated internalization. In fact, in cancer cells, FRα binds to the folic acid conjugate and invaginates to form primarily early endosomes. Early endosomes are characterized by a relatively strong reducing microenvironment, which easily breaks the disulfide bond, releasing vinblastine, which is relatively lipophilic and can freely diffuse into the cytoplasm to exert its antiproliferative/cytotoxic action. Conversely, as described above, FRα serves to salvage folates from the nascent urine in the kidneys, which are transported across the kidney epithelium via transcytosis to the blood stream [33]. This process prevents the release of vinblastine from the receptor and its accumulation in the cytoplasm of proximal tubule cells, thereby minimizing its nephrotoxicity.

In parallel to folate-based chemotherapy, folic-acid-based radioconjugates have been developed for the initial purpose of nuclear imaging-based stratification of FRα-positive tumors to select the patients that might benefit the most from folate-conjugated chemotherapy [110]. Shortly thereafter, folate radioconjugates began to also be considered for tumor-targeted radionuclide therapy that could overcome the main limitation of folate-conjugated chemotherapy, to which only cancer cells overexpressing FRα are sensitive. Folate radioconjugates generally also affect neighboring cells, extending the cytotoxic effect to the fraction of surrounding cancer cells with low or no FRα expression. Unfortunately, a number of studies in animals showed that folic acid radioconjugates are rapidly cleared from the blood, with the tendency to accumulate in the kidneys and little uptake by tumor cells. Moreover, unlike folic acid-conjugated drugs, the radioconjugates do not require cleavage from the receptor to be activated, thereby harming the kidney [111,112]. A number of strategies have been tested in animals with the aim of increasing the tumor uptake as well as limiting the renal accumulation. Thus far, the most promising results have been obtained by injecting pemetrexed one hour prior the treatment with the radioconjugate: the tumor-to-background ratio of radiofolates was increased and the kidney accumulation reduced. Pemetrexed, unlike other antifolates, binds tightly to and dissociates slowly from FRα, acting as a relatively strong competitor of folic acid for the receptor [113]. Nonetheless, the tumor-to-kidney radioactivity ratio is still not acceptable to foresee a therapeutic application of radiofolates in the near future [114,115,116,117,118].

## 6. Folic Acid-Bound Drugs for the Treatment of Polycystic Kidney

Autosomal dominant polycystic kidney disease (ADPKD) is the most common human monogenetic disease with a prevalence of 1–2 cases per one thousand individuals. The most common renal complication of ADPKD is hypertension followed by bacterial infections of the urinary tract and nephrolithiasis (kidney stones). Patients with ADPKD often develop renal failure over time, requiring hemodialysis and/or transplantation. The majority of ADPKD cases are caused by mutations in the polycystic kidney disease 1 gene (*PKD1)*, which encodes Polycystin-1, a large plasma membrane protein involved in cell-cell or cell-matrix interactions. Mutations in the polycystic kidney disease 2 (*PKD2)* gene account for the remaining cases of ADPKD. Polycystin-2 is also a membrane protein and can act as a cation channel, and may be regulated by Polycystin-1. Over the past decades some light has been shed on the molecular mechanisms that abnormally regulate PKD, which are potential targets for therapy. For a detailed overview of polycystic kidney disease, we refer to the recent work of Ghata and Cowley [119]. 

From a therapeutic standpoint, reducing the uncontrolled cellular proliferation that characterizes PKD is considered the most promising strategy. However, there is currently no therapy approved in the United States and only the vasopressin receptor antagonist Tolvaptan is approved outside of the United States. Tolvaptan blocks vasopressin-mediated cyclic AMP (cAMP) production, which promotes cell proliferation of renal cystic epithelia [119,120,121]. Another key pathway for cell proliferation during PKD is the mammalian target of rapamycin (mTOR) pathway. Administration of the mTOR inhibitors rapamycin or everolimus effectively reduces renal cystogenesis in different PKD animal models [122,123,124]. Unfortunately, neither rapamycin nor everolimus were effective in patients with ADPKD [125,126,127]. 

The negative outcome in clinical trials, despite the efficacy observed in the preclinical phase, has been explained with the relatively lower doses used clinically than the much higher doses administered to animals. Systemic toxicity of high doses of mTOR inhibitors has often been observed in transplant patients. While the doses of rapamycin commonly used for immunosuppression are insufficient for significant inhibition of mTOR in the human kidney, higher dose regimens do not appear to be feasible without targeting the drug to the kidney in order to achieve high local concentrations while avoiding, or significantly reducing, systemic side effects. The limited expression of FRα in most adult tissues, combined with the high expression in renal cyst-lining cells of human ADPKD and PKD mouse models, offer an intriguing approach for the optimization of the treatment of polycystic kidney disease. Rapamycin conjugated to folic acid contains one folic acid–targeting moiety with a hydrophilic pentapeptide spacer extended off its gamma glutamyl residue, and coupled to the rapamycin drug payload via a disulfide-based, per self-immolative linker system. In vitro, folate-conjugated rapamycin inhibited mTOR activity in a dose- and folate receptor-dependent manner. Treatment of a PKD mouse model with folate-conjugated rapamycin inhibited mTOR in the kidney, resulting in a strong attenuation of renal cysts growth and preserved renal function, without obvious off-target mTOR activity inhibition [128]. In a head-to-head comparison, both folate-conjugated rapamycin and unconjugated rapamycin inhibited renal cyst growth, mTOR activation, cell cycling, and fibrosis in a polycystic kidney disease mouse model, with folate-conjugated rapamycin sparing the other organs from its immunosuppressive effect [129].

More recently, a folate–dactolisib conjugate for targeting tubular cells in polycystic kidneys has been developed. Dactolisib is a dual inhibitor of phosphatidylinositol 3-kinase (PI3K) and mTOR. In this case, dactolisib was linked to a thiol-containing spacer (e.g., PEG5-Cys) and an ethylenediamine platinum(II) linker. The conjugate was internalized efficiently by immortalized proximal tubule epithelial cells expressing FRα (HK-2 cells) and the intracellular accumulation was reduced by co-incubation with an excess of folic acid, demonstrating a protein-mediated uptake. In these cells, nanomolar extracellular concentrations of the conjugate were able to inhibit PI3K and mTOR pathways. After intraperitoneal administration, the conjugate accumulated extensively in kidneys of a polycystic kidney disease mouse model with minimal uptake in other organs. Unfortunately, no endpoints for efficacy and toxicity were measured in this first preclinical study [130]. 

## 7. Conclusions

Renal reabsorption is important for folate homeostasis and is mainly mediated by FRα. It is known that, under folate deficiency, other endocytic or transporter-mediated processes come into play for the recycling of folates. Thus, we wonder how this workload is redistributed in the kidneys of those individuals that have supraphysiological plasma levels of folic acid and 5-methylTHFfolate resulting from folic acid supplementation and/or fortification of food products. Moreover, the roles of the other folate-specific transporters (PCFT and RFC) in the kidney remain elusive and require further in vivo studies, particularly, in light of the application of folate-conjugate drugs and antifolates in the treatment of various cancers, chronic inflammatory disorders (e.g., rheumatoid arthritis) [131], and the polycystic kidney. For instance, it would be important to understand the role of PCFT in renal folate handling in the pcft^−/−^ mouse model supplemented with folates to ensure normal folate blood levels. The generation of a kidney-specific pcft knockout would likewise contribute to elucidating the role of this transporter in renal folate reabsorption. What impact do the low-affinity, high-capacity folate transporters have on folate homeostasis? Furthermore, what would their respective impact be on folate-conjugate therapies, particularly in those where free folates are additionally administered, saturating or competing for binding to the high-affinity FRα receptor? Further studies on the kidney are also required for the development of folate-guided radiotherapy. Despite the great potential, the rapid clearance and the high accumulation in the renal parenchyma render radiofolates unusable. A better understanding of the pharmacology of these folate-based drugs is required to develop therapeutic strategies that would couple a high tumor accumulation with rapid clearance and low renal accumulation in order to limit the nephrotoxicity. Finally, further studies need to be done on the FRα function in renal cysts as cyst formation is known to alter cell polarity, which could, in turn, reduce the accessibility of the receptor to the folate conjugate [132].
